# A *Klebsiella pneumoniae* DedA family membrane protein is required for colistin resistance and for virulence in wax moth larvae

**DOI:** 10.1038/s41598-021-03834-3

**Published:** 2021-12-21

**Authors:** Vijay Tiwari, Pradip R. Panta, Caitlin E. Billiot, Martin V. Douglass, Carmen M. Herrera, M. Stephen Trent, William T. Doerrler

**Affiliations:** 1 Department of Biological Sciences, Louisiana State University, Baton Rouge, LA USA; 2 Department of Infectious Diseases, College of Veterinary Medicine, University of Georgia, Athens, GA USA

**Keywords:** Genetics, Microbiology

## Abstract

Ineffectiveness of carbapenems against multidrug resistant pathogens led to the increased use of colistin (polymyxin E) as a last resort antibiotic. A gene belonging to the DedA family encoding conserved membrane proteins was previously identified by screening a transposon library of *K. pneumoniae* ST258 for sensitivity to colistin. We have renamed this gene *dkcA* (*dedA* of *Klebsiella* required for colistin resistance). DedA family proteins are likely membrane transporters required for viability of *Escherichia coli* and *Burkholderia spp.* at alkaline pH and for resistance to colistin in a number of bacterial species. Colistin resistance is often conferred via modification of the lipid A component of bacterial lipopolysaccharide with aminoarabinose (Ara4N) and/or phosphoethanolamine. Mass spectrometry analysis of lipid A of the *∆dkcA* mutant shows a near absence of Ara4N in the lipid A, suggesting a requirement for DkcA for lipid A modification with Ara4N. Mutation of *K. pneumoniae dkcA* resulted in a reduction of the colistin minimal inhibitory concentration to approximately what is found with a Δ*arnT* strain. We also identify a requirement of DkcA for colistin resistance that is independent of lipid A modification, instead requiring maintenance of optimal membrane potential. *K. pneumoniae* Δ*dkcA* displays reduced virulence in *Galleria mellonella* suggesting colistin sensitivity can cause loss of virulence.

## Introduction

Colistin (polymyxin E) is a last resort antibiotic for treatment of infections caused by Gram-negative pathogenic bacteria such as *Klebsiella pneumoniae*^1^. Unfortunately, colistin-resistant mutants have emerged, leading to the spread of high-risk clones that are resistant to all antimicrobial agents, defined as ‘pan-drug resistant’. It is estimated that by 2050 antimicrobial resistance will cause 10 million deaths annually if no action is taken^2^. This crisis is worsened by a scarcity of new antimicrobials, especially against Gram-negative pathogens and worldwide dissemination of infections associated with globalization^3^. Compounds that target colistin resistance are attractive targets for use as adjuvants and, potentially, as antivirulence drugs^4–7^.

The DedA family is a highly conserved membrane protein family that remains poorly characterized, belonging to the “SNARE-associated PF09335” family of proteins (PFAM 34.0), with 29,230 individual sequences across 7915 species. We have characterized members of the DedA family in *Escherichia coli, Borrelia burgdorferi, Burkholderia thailandensis* and *Burkholderia glumae*^8–19^. The DedA family includes *E. coli* YqjA and YghB that are putative proton dependent transporters that are required for normal cell division^10,11,13^, alkaline tolerance^16,20^ and resistance to a number of antibiotics and biocides^15^. Both YqjA and YghB possess essential membrane embedded charged amino acids^14,15^ that are present in proton-dependent transporters belonging to the major facilitator superfamily and other transporter families^21–29^. While the reasons for this are unclear, DedA family proteins are required for polymyxin and/or antimicrobial peptide resistance of *Salmonella enterica*^30^, *Neisseria meningitidis*^31^, *Klebsiella pneumoniae*^6^ and *B. thailandensis*^17,18^ and *B. glumae *^19^. DedA has been identified as a member of the colistin “secondary resistome” of multidrug resistant *Klebsiella pneumoniae* using TNseq and as such may be a target of helper drugs designed to restore the efficacy of colistin toward this and possibly other bacterial pathogens^6^.

Colistin acts in a manner mechanistically similar to the CAMPS (cationic antimicrobial peptides) of the innate immune response by binding to and disrupting negatively charged bacterial membranes leading to cell lysis and death^32^. CAMPs are found in all kingdoms of life where they play roles in immune defense, predation and competition^33,34^. Gram-negative pathogens, including *K. pneumoniae*, can acquire resistance to colistin by modification of outer membrane lipopolysaccharides (LPS) with cationic substituents resulting in reduced affinity to the polycationic colistin^35^. Common mechanisms involve expression of enzymes, including aminoarabinose (Ara4N) transferase (ArnT) and phosphoethanolamine transferase (EptA) which transfer aminoarabinose and phosphoethanolamine, respectively, to LPS lipid A. Together, these amine-containing groups neutralize the negative charge of lipid A disrupting colistin binding^35^. The discovery of plasmid associated *eptA* homologues (termed *mcr* for “mobile colistin resistance”) genes raise concern about possible spread of colistin resistance via horizontal gene transfer^36–38^.

Cytoplasmic synthesis and inner membrane transport of undecaprenyl phosphate-α-L-Ara4N precedes periplasmic modification of lipid A with Ara4N (1). The transport of this lipid-linked intermediate is carried out by EmrE-like transporters designated ArnE and ArnF (2). Proton motive force (PMF)-dependent transport of EmrE substrates such as methyl viologen and ethidium bromide is compromised in an *E. coli* mutant with deletions of two DedA family members (3). It is therefore possible that transport of undecaprenyl-P-α-L-Ara4N is inefficient in certain DedA family mutants resulting in production of lipid A that lacks Ara4N and colistin sensitivity. We have renamed *K. pneumoniae dedA dkcA* (*dedA* of *Klebsiella* required for colistin resistance). In this work, we analyzed a Δ*dkcA* mutation in virulent, multidrug resistant *K. pneumoniae* ST258 and demonstrate an altered lipid A structure that is almost completely lacking Ara4N. In addition, we report that DkcA is required for lipid A modification-independent colistin resistance, instead requiring DkcA for optimization of membrane potential. Finally, we demonstrate DkcA is required for virulence in *Galleria mellonella* suggesting that colistin sensitivity can cause loss of virulence in vivo, consistent with previous studies demonstrating the importance of membrane polarization^39^ and lipid A modifications^40^ for virulence. This work supports the lipid A modification pathways^4,7^ and DkcA^6^ as potential targets of colistin adjuvants and anti-virulence drugs.

## Results

**Figure 1:**
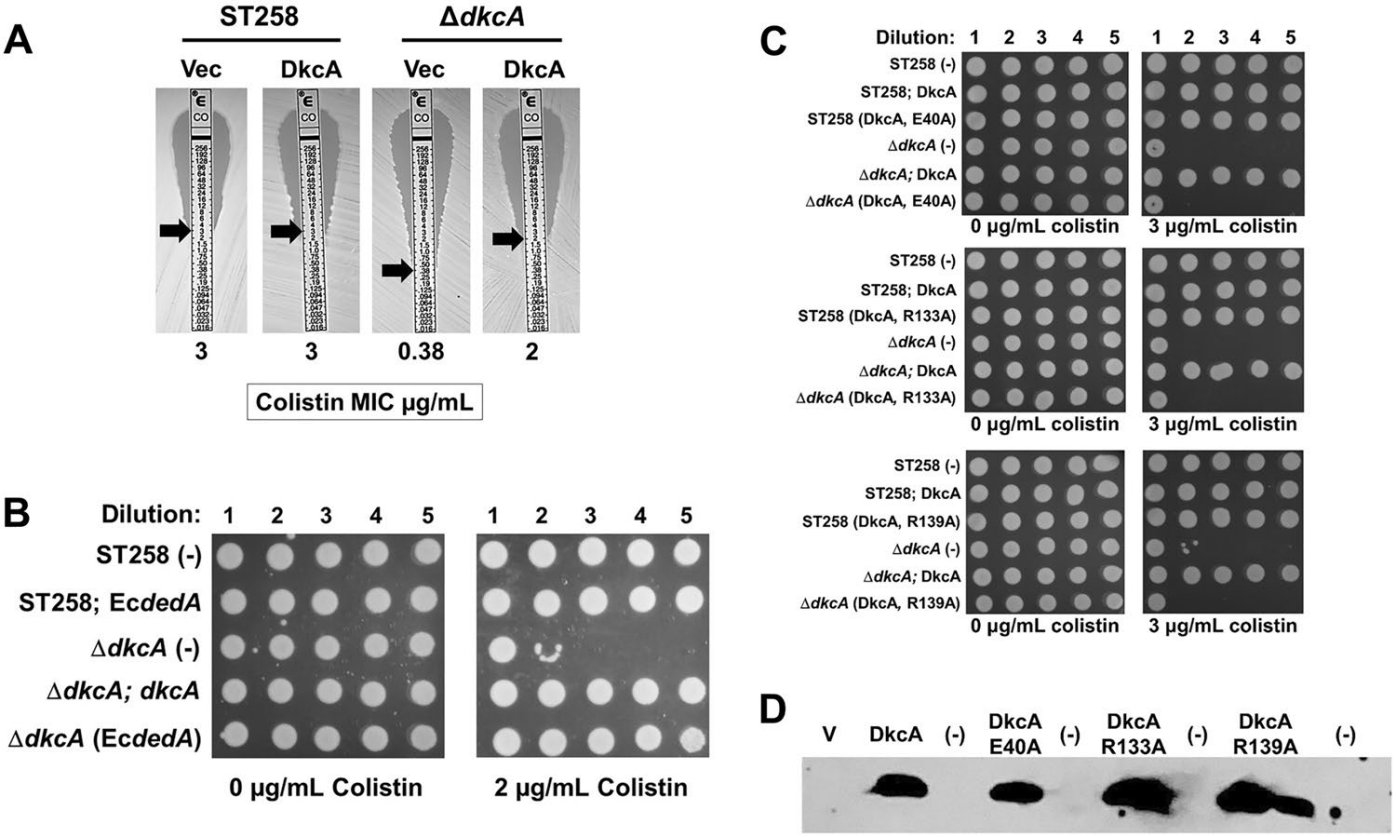
Sensitivity of *K. pneumoniae* ST258 Δ*dkcA* to colistin and requirement of DkcA conserved, charged amino acids. **(A)** MIC was determined using colistin E-test strips for *K. pneumoniae* wild type and Δ*dkcA* harboring control vector (-) or vector expressing *dkcA*. Approximate MIC is indicated with arrows and numerically below each image. **(B)** 1:10 dilutions of the indicated log-phase grown strains were spotted and grown on MH2 agar medium containing 0 or 2 µg/ml colistin containing 0.1% arabinose. **(C)** Dilutions of mid-log phase grown cells of the indicated strains were spotted on MH2 agar plates containing 0.1% arabinose and 3 µg/ml colistin. **(D)** Expression of *dkcA* and point mutants in membrane fractions as determined by Western blotting with anti-hexahistidine antibody. The cropped image was not manipulated in any way and full-length blot is presented in supplementary material (see Supplementary Fig. S4 online). Abbreviations: V = vector control; (-) is lane with no protein loaded. Ten µg of membrane protein was loaded per lane and strains were grown in the presence of 50 µg/ml apramycin and 0.1% arabinose.

Construction of a *K. pneumoniae* ST258 Δ*dkcA* mutant and a complementing pBAD-based plasmid (engineered with an apramycin-resistance marker to allow for selection in MDR *K. pneumoniae*) expressing *dkcA* with an N-terminal His-tag, has been described previously^6^. Measurement of the colistin minimal inhibitory concentration (MIC) using E-strips demonstrates that *K. pneumoniae* Δ*dkcA* is nearly eight times more sensitive to colistin than wild type (Fig. 1A) and that expression of *K. pneumoniae dkcA* or *E. coli dedA* from a plasmid restores sensitivity of the mutant to near wild type levels (Fig. 1B) consistent with our previous observation^6^.

While *K. pneumoniae* DkcA is ~ 90% identical to *E. coli* DedA, most of our work with the *E. coli* DedA family has been with YqjA and YghB proteins because of the striking phenotypes of the double mutant strain^11–13,15^, and the Δ*yqjA* strain^16^. We have also characterized *dkcA* homologues from *B. thailandensis* and *B. glumae* named *dbcA* (*dedA* of *Burkholderia* required for colistin resistance), which are required for high-level colistin resistance in these species^17–19^. *K. pneumoniae* DkcA shares only ~ 30% amino acid identity to *E. coli* YqjA, YghB, and *Burkholderia* DbcA proteins but still possesses membrane-embedded charged amino acids E40, R133 and R139 in similar locations as found in their YghB/YqjA counterparts that we have shown are critical for function (see Supplementary Fig. S1 online for amino acid alignment)^14,15^. Therefore, we sequentially changed E40, R133 and R139 each to alanine and determined if the mutant proteins were capable of complementing the colistin sensitivity of the Δ*dkcA* strain. We found that all three mutants were inactive in this assay, demonstrating the importance of these charged amino acids (Fig. 1C). All proteins were found to be membrane associated, ruling out misfolding of the mutant proteins (Fig. 1D). These findings suggest that *K. pneumoniae* DkcA may function as a proton-dependent membrane transporter, similar to other members of the DedA family.

**Figure 2:**
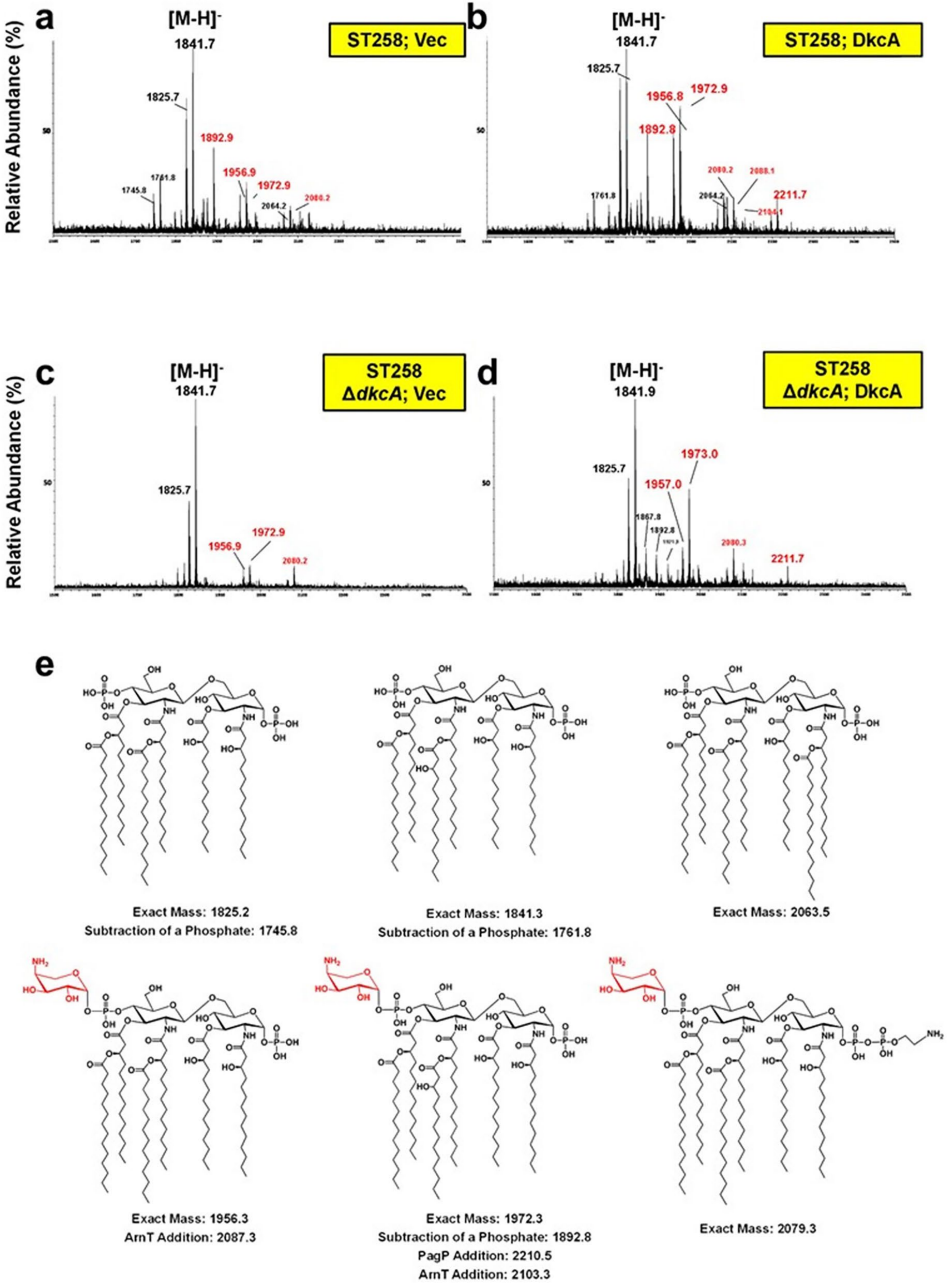
Mass spectrometry of lipid A isolated from *K. pneumoniae* strains. **(a–d)** Lipid A extracted from the indicated strains was analyzed using a MALDI-TOF mass spectrometer (ABI 4700 Proteomic Analyzer) in the negative-ion linear mode. Species modified with Ara4N are labeled in red font. **(e)** Structures of each observed species.

Since lipid A modifications contribute to colistin resistance in numerous Gram-negative species including *Klebsiella pneumoniae*^41,42^, the lipid A structure of wild type *K. pneumoniae* ST258 and Δ*dkcA* grown in LB media was analyzed using mass spectrometry. In wild type *K. pneumoniae* (Fig. 2a), major species are hexa-acyl lipid A (m/z: 1825.2) and its hydroxylated derivative (m/z: 1841.3). In addition, several species containing Ara4N were observed (designated by red font in Fig. 2; m/z: 1892.8, 1956.3, 1972.3, and the phospoethanolamine/Ara4N modified 2079.3). A similar pattern of lipid A species was observed in wild type and Δ*dkcA* expressing *dkcA* from a plasmid (Fig. 2b and d). However, in the Δ*dkcA* strain harboring the control vector, Ara4N modified species of lipid A were substantially decreased by MS (Fig. 2c), similar to what was found with a *Burkholderia thailandensis* Δ*dedA* strain^18^. Levels of the dual modified Ara4N/phosphoethanolamine species m/z 2079.3 were low but detectable at similar levels in all strains, suggesting the phosphoethanolamine modified species constitutes a minor portion of the total lipid A and is unaffected by the Δ*dkcA* mutation. See Fig. 2e for structures of these species. This observation in combination with the marked sensitivity to colistin is noteworthy and prompted us to analyze the *K. pneumoniae* DkcA protein and Δ*dkcA* strain in more detail.

**Figure 3:**
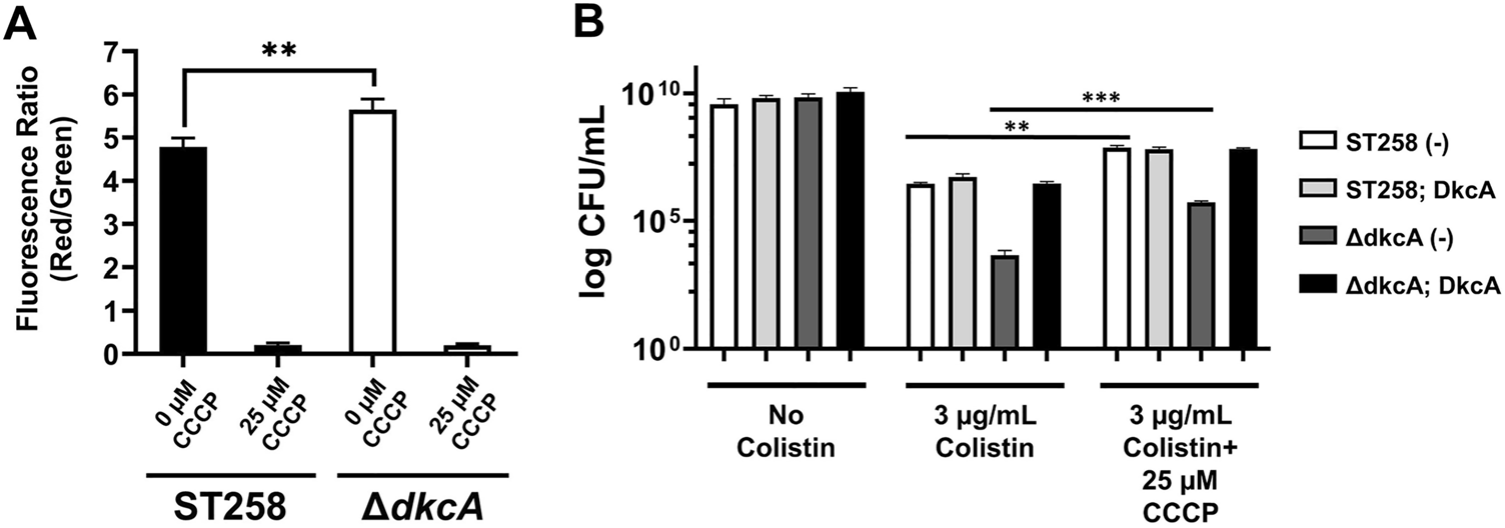
A role for DkcA in maintenance of membrane potential and colistin resistance of *K. pneumoniae*. **(A)** Membrane hyperpolarization of Δ*dkcA* compared to ST258. ST258 and Δ*dkcA* mutant treated with 25 µM CCCP for 30 min showing loss of Δψ were used as the control. **(B)** Partial restoration of colistin resistance of ∆*dkcA* by CCCP. Cells grown to OD_600_ of 0.8 in MH2 media containing 50 µg/ml Apr, 0.1% arabinose were diluted ten-fold and incubated in the same media alone or exposed to 3 µg/ml colistin or 3 µg/ml colistin plus 25 µM CCCP for 45 min at 37 °C. After exposure, cells were washed with fresh MH2 medium twice, resuspended in MH2 and CFU/ml was determined by plating serial dilutions. Each experiment was repeated three times. Statistical significance was calculated by unpaired Student’s t-test. **: *p* < 0.01. ***: *p* < 0.001.

In our previous work analyzing functions of DedA family members from *E. coli*, *B. thailandensis*, and *B. glumae*, we observed that mutation of DedA family members causes alterations in the membrane proton motive force. These changes were somewhat inconsistent in that *E. coli* and *B. thailandensis* mutants were somewhat depolarized^12,18^, but a *B. glumae* mutant strain was hyperpolarized^19^. This suggests that DedA family proteins may function in different manners in optimizing membrane potential depending upon the gene and organism in which they are found. Membrane potential was measured in *K. pneumoniae* ST258 and Δ*dkcA* and we found that the Δ*dkcA* strain was slightly but significantly hyperpolarized (Fig. 3A). We were unable to complement the membrane hyperpolarization of Δ*dkcA* with pBAD*dkcA* at various levels of arabinose induction due to the observed effect of arabinose on membrane potential even in the wild type strain (data not shown). To determine if this hyperpolarization can result in colistin sensitivity, cells were briefly exposed to colistin (45 min) in the presence or absence of 25 µM CCCP, a proton ionophore, to cause membrane depolarization. Cell survival for all four strains was low in the presence of colistin alone. It was found that a brief preexposure to CCCP could partially rescue cells, both wild type and Δ*dkcA*, from the lethal effects of colistin (Fig. 3B). This result suggests that colistin may require optimum membrane potential as part of its mechanism of action. This hypothesis was tested in a series of experiments.

**Figure 4:**
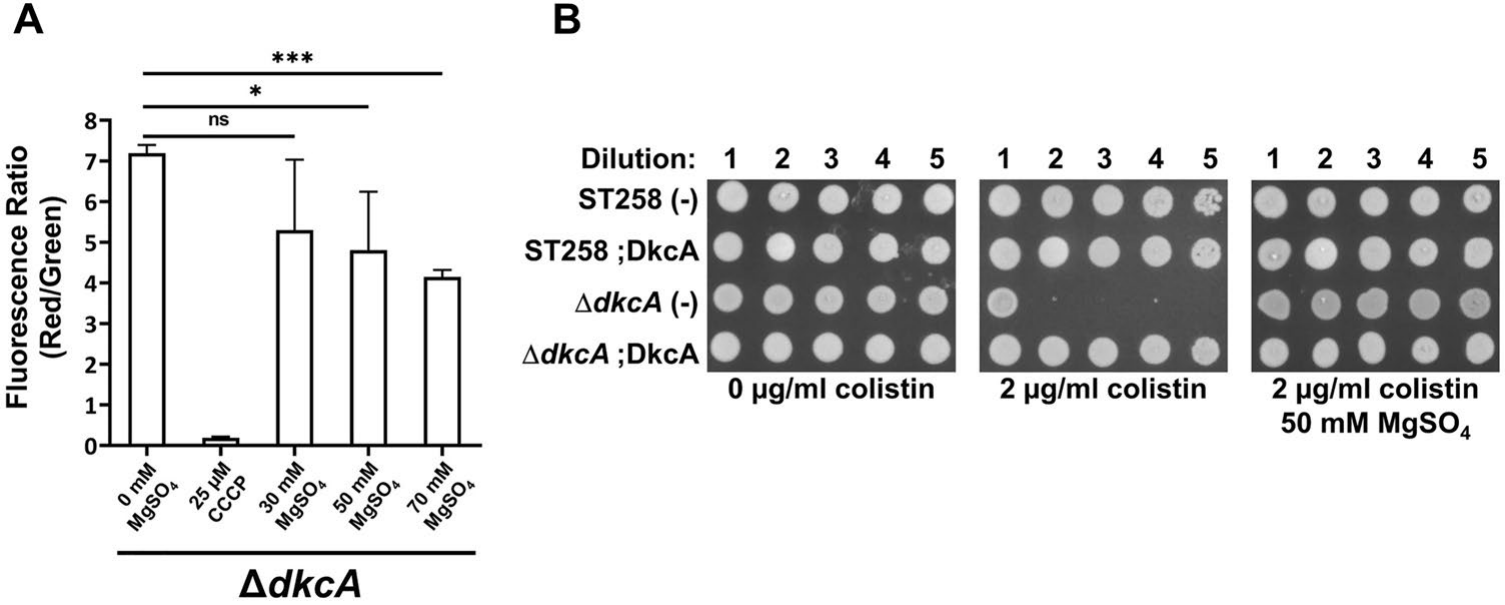
**(A)** Mg^++^ alleviates hyperpolarization of ∆*dkcA*. Mg^++^ at indicated concentrations were added in the media, cells were grown to OD_600_ of 0.8 and membrane potential was determined. ST258 and Δ*dkcA* mutant treated with 25 µM CCCP for 30 min were used as controls. Each experiment was repeated three times. Statistical significance was calculated by unpaired Student’s t-test. *: *p* < 0.05, ***: *p* < 0.001, ns: not significant. **(B)** Complementation of colistin sensitivity of Δ*dkcA* by Mg^++^. 1:10 dilutions of indicated strains were spotted and grown on LB media containing 0 or 2 µg/ml colistin with 50 µg/ml Apr, 0.1% arabinose and indicated concentrations of magnesium sulphate. Plates were incubated at 37 °C for 24 h.

Magnesium influx has been reported to alleviate hyperpolarization associated with ribosomal stress in *Bacillus subtilis*^43^. In contrast, exposure to sublethal ribosome targeting antibiotics such as spectinomycin can increase the number of cells that exhibit membrane hyperpolarization^43^. Therefore, we tested the ability of extracellular Mg^++^ to rescue *K. pneumoniae* strains from the lethal effects of colistin and sub-MIC concentrations spectinomycin to sensitize strains to colistin. While Mg^++^ is known to protect Gram negative bacteria from the effects of colistin by competing for binding with negatively charged sites on LPS^44^, it has also been shown to contribute to polymyxin resistance independently of LPS and the PhoPQ regulon by its ability to depolarize the membrane^39^. We measured the effects of Mg^++^ on membrane potential and colistin sensitivity of *K. pneumoniae* ST258 and Δ*dkcA*. Growth in the presence of 50 mM or higher of Mg^++^ causes membrane depolarization of *K. pneumoniae* Δ*dkcA* (Fig. 4A). Mg^++^ at 50 mM completely restores colistin resistance to this mutant (Fig. 4B) under conditions predicted to repress expression of the PhoPQ operon^45^. These results collectively show that alleviation of membrane hyperpolarization of wild type cells or Δ*dkcA* strain with Mg^++^ results in increased colistin resistance, again supporting a role of proper regulation of membrane potential as critical for colistin resistance, likely independently of any alterations to the structure of lipid A.

**Figure 5:**
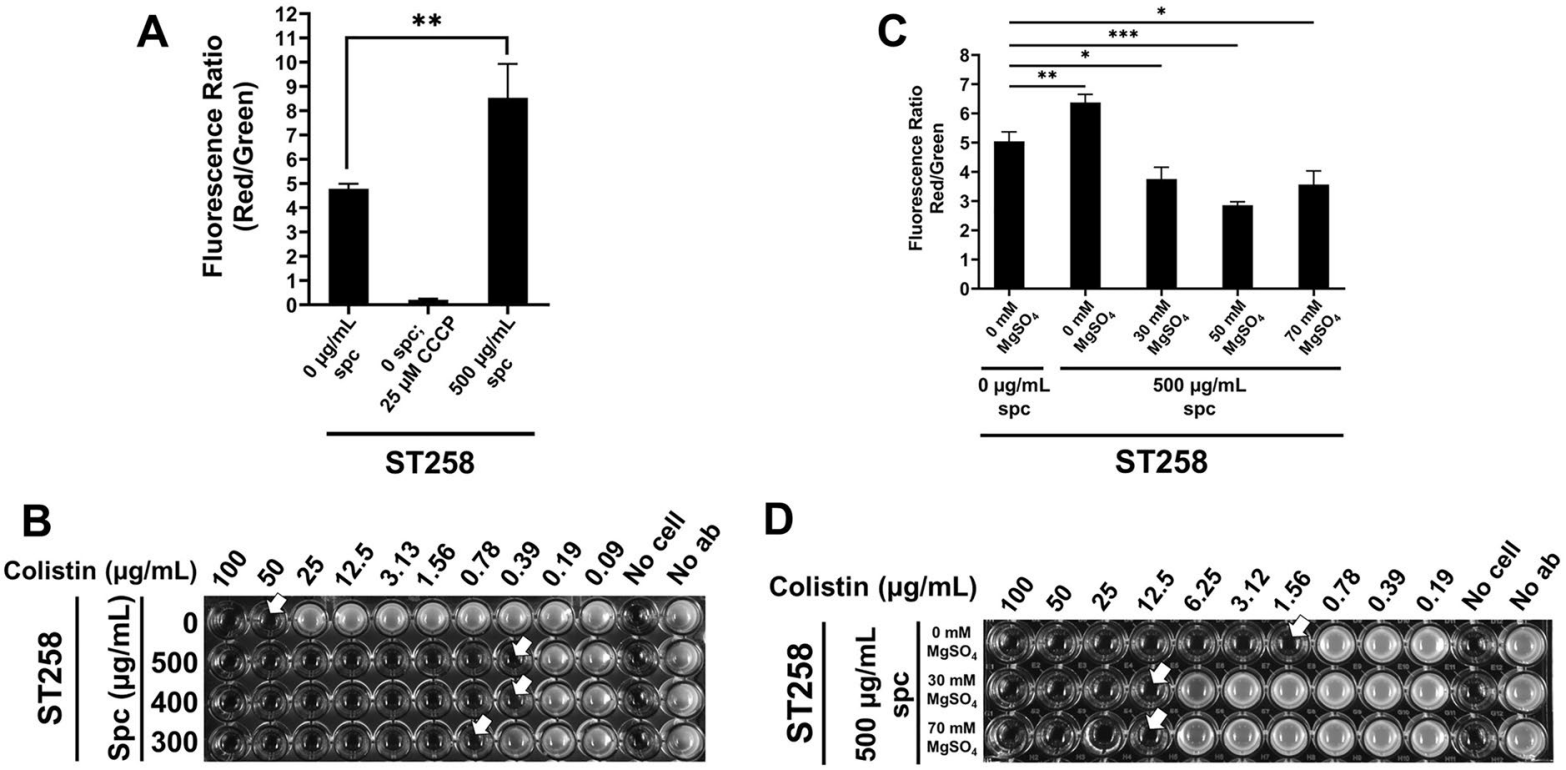
Chemical alteration of membrane potential of *K. pneumoniae* by spectinomycin and its effect on colistin resistance. **(A)** Membrane hyperpolarization of ST258 by spectinomycin. Spectinomycin (500 µg/mL) was added at the start of culture in LB media, and the cells were grown to OD_600_ = 0.6 and membrane potential was determined. ST258 treated with 25 µM CCCP for 30 min was used as a control. **(B)** Spectinomycin exposure increases colistin sensitivity of ST258. Colistin MIC was determined at indicated concentrations of spectinomycin. Approximate MIC values are indicated with white arrows. **(C)** Mg^++^ alleviates the hyperpolarization of membrane potential in ST258 caused by spectinomycin. Spectinomycin (500 µg/mL) and indicated concentration of Mg^++^ was added at the start of culture in LB media, and the cells were grown to OD_600_ = 0.6 and membrane potential was determined. **(D)** Mg^++^ reverses spectinomycin mediated reduction of colistin MIC. Colistin MIC was measured at indicated concentration of Mg^++^ for ST258 treated with 500 µg/ml spectinomycin. Approximate MIC values are indicated with white arrows. **5(A)** and (**C**), each bar represents the average value and standard deviation from a triplicate experiment. Each experiment was repeated three times. Statistical significance was calculated by unpaired Student’s t-test. *: *p* < 0.05. **: *p* < 0.01. ***: *p *< 0.001.

We then measured the effect of spectinomycin-induced hyperpolarization on the colistin sensitivity of *K. pneumoniae* ST258. We first determined the spectinomycin MIC of the strain to be ~ 4000 µg/ml (see Supplementary Fig. S2 online). Exposure of wild type *K. pneumoniae* ST258 to a sub-MIC concentration of 500 µg/ml spectinomycin causes hyperpolarization of membrane potential (Fig. 5A). Spectinomycin treatment of ST258 also causes increased colistin sensitivity, decreasing the colistin MIC by ~ 100 fold (Fig. 5B). This indirectly shows hyperpolarization of membrane potential may be responsible for increased colistin sensitivity and again implies that maintenance of optimal membrane potential is required for colistin resistance. This is also consistent with the observation above that depolarization using a brief exposure to CCCP can partially rescue cells from the lethal effects of colistin (Fig. 3B). Growth in the presence of 30 mM or higher of Mg^++^ causes membrane depolarization of *K. pneumoniae* ST258, even when hyperpolarized by exposure to spectinomycin (Fig. 5C). Mg^++^ also causes significant eightfold increase of the colistin MIC observed in the presence of spectinomycin (Fig. 5D), again showing a strong correlation between membrane polarization and colistin sensitivity.

**Figure 6:**
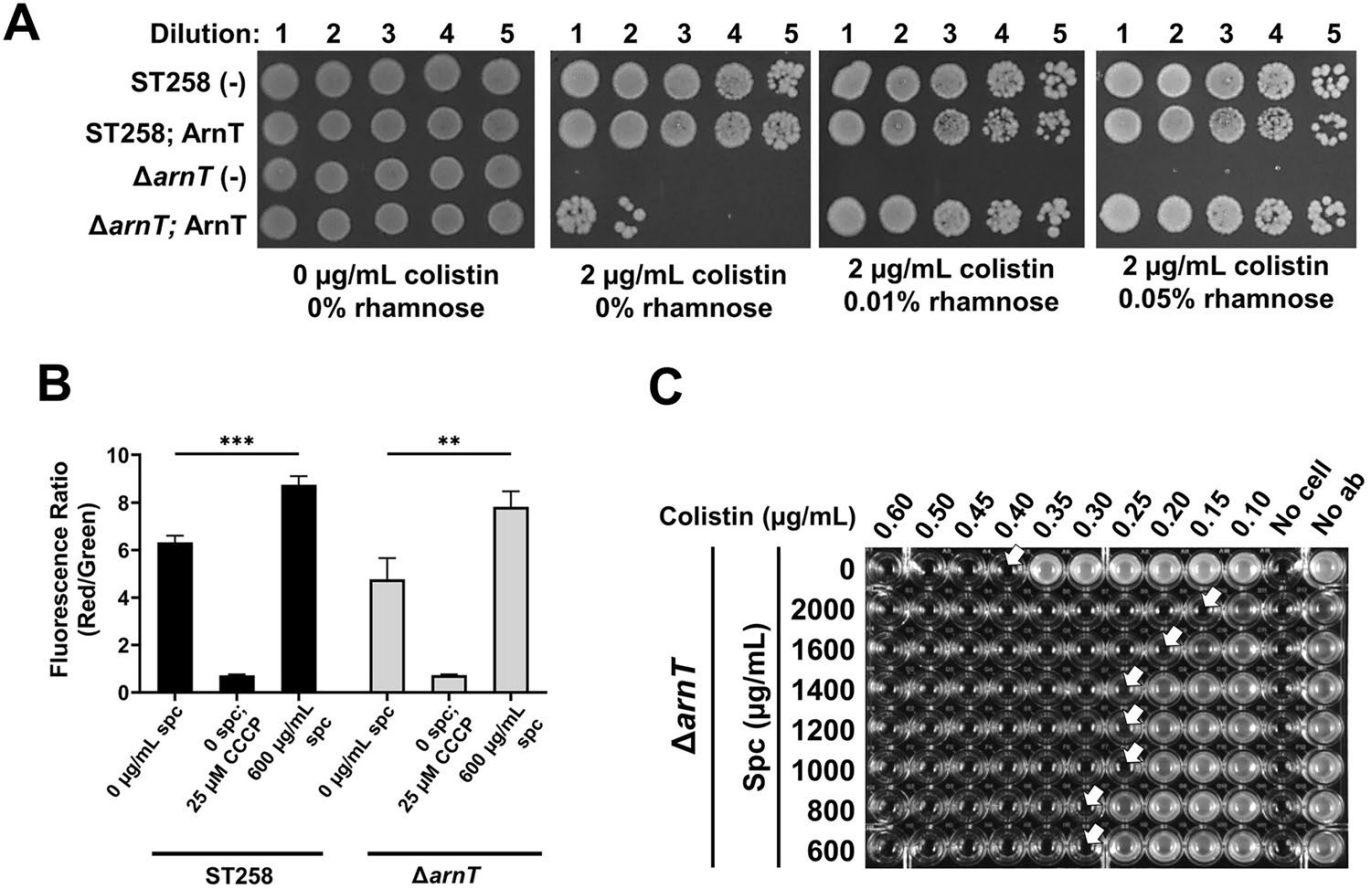
Spectinomycin mediated reduction of colistin MIC is independent of lipid A modification with Ara4N. **(A)** Sensitivity of *K. pneumoniae* ST258 Δ*arnT* to colistin. Dilution of cultures of indicated strains were spotted and grown on LB agar medium containing 0 or 2 µg/ml colistin containing indicated rhamnose concentrations. **(B)** Membrane hyperpolarization of ST258 and Δ*arnT* by spectinomycin treatment. ST258 and Δ*arnT* treated with 25 µM CCCP for 30 min were used as controls. Each experiment was repeated three times. Statistical significance was calculated by unpaired Student’s t-test. **: *p* < 0.01. ***: *p* < 0.001. **(C)** Spectinomycin treatment further sensitizes Δ*arnT* towards colistin. Colistin MIC was measured at indicated spectinomycin concentrations. Approximate MIC values are indicated with white arrows.

To explore this question further and to understand if the effects of membrane polarization are independent of lipid A structure, we constructed an in-frame *K. pneumoniae* ST258 Δ*arnT* strain (see Supplementary Fig. S3 online) along with a complementing plasmid (Table 1) and performed similar measurements. ArnT is the Ara4N transferase required for periplasmic modification of lipid A with Ara4N and colistin resistance^46–48^. The Δ*arnT* strain is sensitive to colistin in the absence of complementation (Fig. 6A). Spectinomycin treatment of *K. pneumoniae* Δ*arnT* causes hyperpolarization of membrane potential to a similar extent as that seen in *K. pneumoniae* ST258 (Fig. 6B). While Δ*arnT* is exquisitely sensitive to colistin (MIC ~ 0.4 µg/ml), exposure to 2000 µg/ml spectinomycin decreases the MIC by as much as 2.7-fold (Fig. 6C). This observation serves as direct evidence showing hyperpolarization of membrane potential is responsible for increased colistin sensitivity and supports our hypothesis that maintenance of optimal membrane potential is required for colistin resistance independent of lipid A modification.

**Table 1:** Bacterial strains and plasmids used in this study.

Strains	Description	Source or Reference
*Escherichia coli*		
XL1 Blue	*recA*1 *endA1 gyrA96 thi-1 hsdR17 supE44 relA1 lac [F´ proAB lacIqZΔM15 Tn10* (Tet^R^)	Stratagene
SM10	*thi thr leu tonA lacY supE recA::RP4-2-Tc::Mu Km λpir*	^49^
*Klebsiella pneumoniae*		
ST258	MDR *K. pneumoniae* ST258 (RH201207)	^6^
Δ*dkcA*	*K. pneumoniae* ST258; Δ*dkcA*	^6^
Δ*arnT*	*K. pneumoniae* ST258; Δ*arnT*	This study
**Plasmids**		
pBADHisA	Expression vector; *araBAD* promoter, Apr^r^	^6^
pBAD*dkcA*	pBAD expressing wild type *dkcA* with N-terminal His_6_ tag. (Formerly pBAD-Apr^R^-Kpn*dedA*)	^6^
pBADdkcAE40A	pBAD expressing wild type *dkcA* with E40A point mutation and N-terminal His_6_ tag	This study
pBADdkcAR133A	pBAD expressing wild type *dkcA* with R133A point mutation and N-terminal His_6_ tag	This study
pBADdkcAR139A	pBAD expressing wild type *dkcA* with R139A point mutation and N-terminal His_6_ tag	This study
pACBSR-HygR	Arabinose inducible vector consisting of λ-Red recombinase gene with hygromycin resistance cassette	^50^
pIJ773	Template to amplify Apr^R^	^50^
pSCrhaB2	Expression vector; ori_pBBR1_*rhaR, rhaS, P*_rhaB_Tet^R^ *mob* +	^51^
pSCarnT	pSCrhaB2 expressing *arnT*	This study

**Figure 7:**
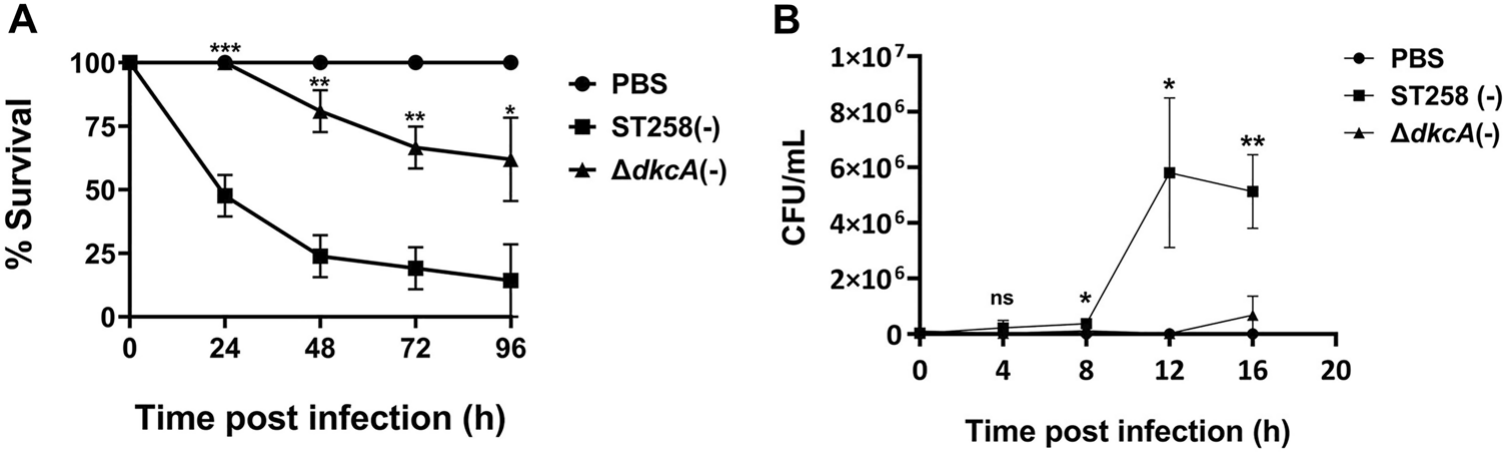
DkcA is required for virulence of *K. pneumoniae* in *Galleria mellonella*. **(A)** Wild-type *K. pneumoniae* ST258 has higher killing rate of larvae compared to Δ*dkcA*. **(B)** *K. pneumoniae* ST258 survival in *G. mellonella* compared to Δ*dkcA*. Data points in each graph represent the average value and standard deviation from three independent determinations from a representative experiment. Each experiment was repeated three times. Statistical significance was calculated by unpaired Student’s t-test. *: *p* < 0.05, **: *p* < 0.01, ***: *p* < 0.001, ns: not significant.

Loss of resistance to colistin suggests mutation of *dkcA* may cause loss of virulence since CAMPS of the innate immune system use the same mechanism to kill bacteria as colistin; i.e. by binding to and disrupting negatively charged bacterial membranes leading to cell lysis and death^32^. In order to measure virulence in vivo, we chose the *Galleria mellonella* moth larvae model due to its moderate costs and ethical considerations. *G. mellonella* has been used successfully to measure virulence of *K. pneumoniae* and many other bacterial pathogens^52^. Using this model, we found the Δ*dkcA* was partially compromised for virulence over the course of the experiment (Fig. 7A). Very few viable bacteria could be recovered from Δ*dkcA* infected larvae, compared to wild type infected larvae, where bacteria could be recovered at 12 and 16 h post infection (Fig. 7B). This suggests that mutations of *dkcA* and other genes^6^ that lead to *K. pneumoniae* colistin sensitivity can also cause loss of virulence and these gene products may also be attractive targets for anti-virulence agents.

## Discussion

Multidrug resistant bacterial infections pose an enormous public safety risk and are a challenge to modern medicine, made worse by a lack of new antimicrobial drugs^2,3^. The emergence of carbapenem-resistant *Klebsiella pneumoniae* is of particular concern and has forced the reintroduction of colistin therapy as a last resort treatment. Colistin is an antimicrobial peptide belonging to the polymyxin family that can cause numerous side effects including nephrotoxicity so dosage must be strictly monitored^53^. The recent emergence of mobilized colistin resistance genes that spread via horizontal gene transfer has further compromised the use of colistin clinically^54^. Helper drugs that can lower the effective dosage of colistin by targeting the “secondary resistome” would be useful therapeutics^6^. Drugs that alter colistin resistance may also result in sensitivity to the cationic antimicrobial peptides of the innate immune system and thus act to target virulence even in the absence of antibiotic administration^4,5^. Potential targets may be *Klebsiella pneumoniae* DkcA or ArnT as each gene deletion strain displays a similar eight-fold reduction in MIC to colistin.

Gram-negative bacteria often become resistant to colistin by activation of genes and pathways that lead to the covalent modification of anionic cell surface lipopolysaccharide with cationic substituents, causing loss of electrostatic binding by the cationic antibiotic^35,55^. In *Klebsiella* and other species, these pathways are controlled by the PhoPQ and PmrAB two-component signaling pathways which lead to modification of lipid A with Ara4N and phosphoethanolamine^55^. While *K. pneumoniae* does harbor a chromosomal homolog of EptA involved in phosphoethanolamine modification of lipid A^56^, our MS analysis suggests this species makes up a minor component of the total lipid A suggesting Ara4N modification is the main contributor to colistin resistance.

**Figure 8:**
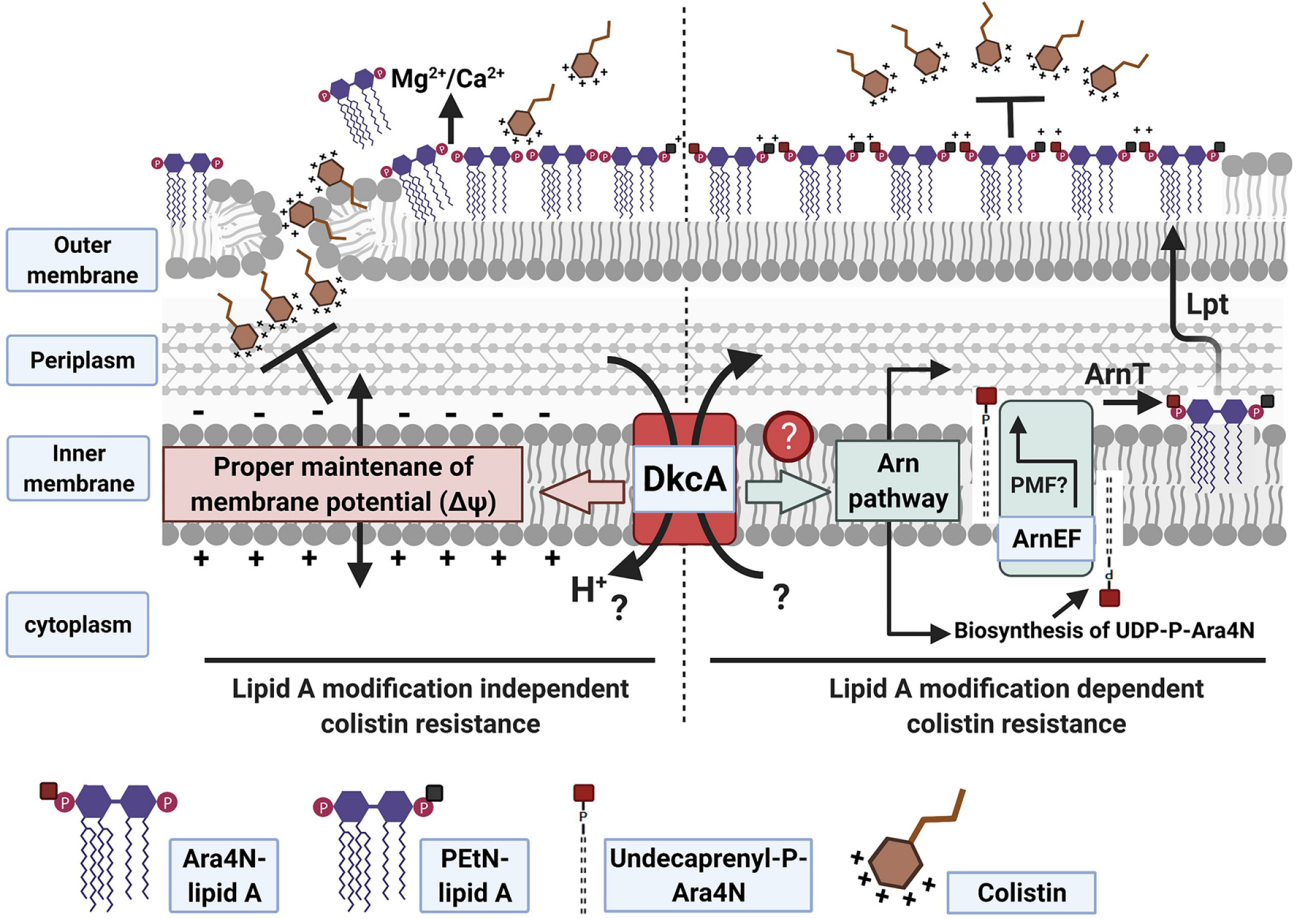
A proposed model of colistin resistance determinants of *Klebsiella pneumoniae* ST258. Both lipid A modification dependent and lipid A modification independent mechanisms may be involved in colistin resistance. DkcA is required for lipid A modification with Ara4N (aminoarabinose) through a yet unidentified mechanism (right side). Lipid A modification with Ara4N increases the overall positive charge of outer membrane and electrostatically repels cationic colistin, contributing to colistin resistance. Lipid A modification with PEtN (phosphoethalonamine) is not affected by the DkcA mutation. DkcA is also involved in proper maintenance of membrane potential contributing to colistin resistance (left side). Mutation of DkcA results in membrane hyperpolarization. By maintaining reversed membrane potential (more positive inside), DkcA could reduce the attraction of cationic colistin molecules towards the membrane and reduce the bactericidal effects of colistin. This is supported by the increased colistin sensitivity by hyperpolarizing agents (spectinomycin) and the reversal of this effect with depolarizing agents (Mg^++^ or CCCP). Thus, an increase in negative membrane potential can electrostatically draw cationic antimicrobial peptides toward the nonpolar inner membrane, the likely site of action of colistin^57^.

Less well characterized is the contribution of membrane potential to colistin sensitivity and resistance. Here, we show that the *K. pneumoniae* Δ*dkcA* is slightly but significantly hyperpolarized and tested the contribution of this hyperpolarization to the observed colistin sensitivity. First, we showed that brief pre-exposure of both wild type and Δ*dkcA* strains to the proton ionophore and depolarizing agent CCCP results in higher survival in the presence of a lethal dose of colistin. The augmented survival against colistin by brief pre-exposure to CCCP is probably due to cytoplasmic acidification by CCCP, consistent with our hypothesis^20^. Next, we showed that treatment with sub-lethal concentrations of the antibiotic spectinomycin causes hyperpolarization of *K. pneumoniae* consistent with what was shown for *B. subtilis*^43^. Importantly, this hyperpolarization was accompanied by an increase in sensitivity to colistin. Therefore, we could mimic the effect of the Δ*dkcA* mutation on both the membrane potential and colistin sensitivity using sub-lethal concentrations of spectinomycin. Relieving the hyperpolarization with magnesium also rescued the sensitivity to colistin. Finally, we showed that the effect of membrane potential on colistin sensitivity was independent of lipid A modification by demonstrating the same effect in a *K. pneumoniae* Δ*arnT* mutant. While Δ*arnT* is sensitive to colistin, consistent with a previous report^6^, exposure to spectinomycin increased this sensitivity while also hyperpolarizing the membrane. Collectively, these results suggest that membrane hyperpolarization can directly lead to colistin sensitivity independently of any changes to the cell surface LPS (Fig. 8).

The DedA family of membrane proteins is widely distributed in nature, found in all kingdoms. Until recently, most of what is known about the family came from studies in *E. coli* due to pleiotropic phenotypes of inactivating mutations. These phenotypes include defects in growth, cell division and hypersensitivity to alkaline pH, antibiotics and membrane penetrating dyes^10–13,15,16^. The correction of these phenotypes by growth in slightly acidic pH and the presence of membrane embedded charged amino acids suggest that members of the DedA family are proton-dependent transporters required for maintenance of optimal PMF^12,14–16^. Our recent studies in *Burkholderia thailandensis* and *B. glumae* also support this hypothesis^17–20^. DedA family proteins have an evolutionary relationship and, indeed may share structural similarity to LeuT, a bacterial homolog of the neurotransmitter:sodium symporter family^58,59^.

While there is no published structure of any DedA family member, modelling of the structure of Human TMEM41b has been carried out which is predicted to possess structural features found in Cl^-^/H^+^ antiporters^60^. The function of human TMEM41B is unknown but it has been identified as being required for autophagosome formation^61–63^ and as a host factor required for infection with flaviviruses as well as SARS-CoV-2^64,65^. While the eukaryotic proteins of the DedA family containing the so-called VTT domain (for VMP, TMEM41, Tvp38)^63^ are distantly related to their bacterial counterparts, and a functional relationship has not been established, the presence of an absolutely conserved glycine residue suggests an evolutionary relationship^66^.

Invertebrate models of bacterial virulence are increasingly common due to lower cost than traditional rodent studies^52^. There are no ethical constraints and their short life span makes them ideal for large-scale studies. While insects do not possess an adaptive immune response, their innate immune response is quite similar to mammals^67^. Insects possess Toll-like receptors, CAMPs, and neutrophil-like cells called hemocytes^68^. There are currently more than 2000 articles published on PubMed on the wax moth *Galleria mellonella* infection model. *Galleria mellonella* has been used to study virulence of *Klebsiella pneumoniae*^40,52,69^ and other bacterial pathogens^70,71^. We show here that *Klebsiella pneumoniae* DkcA is required for virulence in *G. mellonella*, which may be linked to the sensitivity to colistin. These studies are consistent with previous studies demonstrating the importance of optimal membrane potential^39^ and cationic lipid A modifications^40^ for virulence.

In summary, we have shown that the *Klebsiella pneumoniae* DkcA protein is required for colistin resistance in a lipid A-dependent and -independent manner. DkcA displays similarity to proton-dependent membrane transporters and is required for virulence in moth larvae. The bacterial DedA family is evolutionarily conserved and remains an intriguing mystery in terms of its physiological function and it potential for therapeutic targeting.

## Material and methods

### Bacterial growth conditions

*E. coli* cultures were grown on LB medium (1% tryptone, 0.5% yeast extract and 1% NaCl). *Klebsiella pneumoniae* was grown in LB or cation adjusted Mueller–Hinton broth 2 (MH2; Sigma-Aldrich). Antibiotics colistin, ampicillin (Amp; 100 µg/ml), apramycin (Apr; 50 µg/ml) and spectinomycin (at indicated concentrations) were purchased from Sigma-Aldrich or VWR. Cultures were grown at 37 °C unless otherwise indicated. For complementation analysis with pBAD- or pSCrhaB2-derived vectors, arabinose or rhamnose at indicated concentrations was included in growth media. Bacterial strains and plasmids are listed in Table 1.

### Creation of *Klebsiella pneumoniae* Δ*arnT*

The *K. pneumoniae* ST258 *arnT* gene was disrupted using lambda red recombination^50,72^. Bacteria were grown in low salt LB (5 g/L NaCl), pH 8.0 up to the *arnT* disruption step. pACBSR-Hyg^R^ was introduced into ST258 by electroporation and grown at 30 °C. Knockout primers were designed in a way that *arnT_*KO_forward primer has 60 bp homology to the 5’ upstream region and *arnT*_KO_reverse primer has 60 bp homology to the 3’ downstream region of the *arnT* gene (Table 2). An apramycin resistance cassette (Apr^R^) was amplified from pIJ773^50^ using these primers. The linear DNA fragment was purified using QIAquick Gel Extraction Kit (Qiagen). Purified linear DNA was treated with *DpnI* and repurified prior to introduction by electroporation into ST258/pACBSR-Hyg^R^. Following recovery in the absence of antibiotic, the cells were plated on 50 µg/mL Apr. Resistant colonies were screened for colistin sensitivity and gene disruption was verified using PCR primers P1F, P2R, P3F, P4R (Table 2, see Supplementary Fig. S3 online).

**Table 2:** Oligonucleotide primers used in this study.

Primer name	Primer Sequence
SeqpBADdkcAF	GTAACAAAGCGGGACCAAAGC
SeqpBADdkcAR	GGCGTTTCACTTCTGAGTTCG
**Primers used for site-directed mutagenesis study of DkcA protein**	
E40AFW	GTTCTGCGCAACCGGGCTGGTGGTGAC
E40AREV	CCCGGTTGCGCAGAACAGAATCAAAAACAGAATGGCATAAAC
R133AFW	CCTGGCGGCCTTTGTGCCGATAGTCCGAACCTTC
R133AREV	CACAAAGGCCGCCAGGATAATGGTTTTCCCGC
R139AFW	GATAGTCGCAACCTTCGCGCCGTTTGTG
R139AREV	GAAGGTTGCGACTATCGGCACAAAGCGCG
**Primers used to knock out *arnT* from wild type *K. pneumoniae* ST258 background**	
*arnT*_KO_forward	GGGGCGCTAAAGCGCGGCTGGCTTGAGCGCGATGAGGCCCGCGGCGCGCTGTATCTGTTACGAATAAGGGACAGTGAAGAAGGAACACC
*arnT*_KO_reverse	ATAGGTCGGCAGTTTGCCTTTGGCGATACTGAAGAACAGGAACGGCATCGCCACCCAACCCTGCAGGAATTCGATGTGTAGGCTG
**Primers used for the verification of insertion of *arnT* knock out cassette consisting apramycin selection marker**	
P1F	AATATTGAATTCCCTGCTGGACTGCATCCATCG
P2R	CGCTGCGCTTTACATTTGGCAG
P3F	CCTGCCAAATGTAAAGCGCAGC
P4R	ATTAATAAGCTTCAGAATGACAATGCCCACGACGATC
**Primers used for cloning *arnT* into expression vector**	
KparnT1	ATTATACATATGATGAAAAGCATTCGCTATGGCGTCTC
KparnT2	AATATTTCTAGATTATTGCGGCAGGTACTGAAGGAAAAC

### Cloning of *K. pneumoniae* ST258 *arnT*

*K. pneumoniae* ST258 genomic DNA was extracted using Easy-DNA kit (Invitrogen). PCR amplification using Q5 DNA polymerase was performed using KparnT1 and KparnT2 primers (Table 2). For cloning, the stop codon TGA was changed to TAA to prevent modification by Dam methylation. PCR amplification using ST258 genomic DNA generated a linear DNA fragment of 1656 bp. The DNA fragment was gel purified using QIAquick Gel Extraction Kit (Qiagen) and digested with *NdeI* and *XbaI* (New England Biolabs). pScrhaB2^51^ was similarly digested with *NdeI* and *XbaI* and dephosphorylated using Antarctic phosphatase (New England Biolabs). DNA fragments were ligated using Hi-T4 DNA ligase (New England Biolabs). The ligated product was transformed into SM10^73^ and Tet^R^ resistant cells were selected on LB supplemented with 25 µg/mL tetracycline. Cloned *arnT* was expressed from the resulting plasmid pSC*arnT* using 0.01 or 0.05% rhamnose.

### Site-directed mutagenesis

Site-specific mutants were created by using a previously published protocol^74^. The primers carrying the site-specific mutations (Table 2) were used in a PCR reaction to amplify a vector containing the wild type gene**.** The PCR product was then digested with *Dpn1* and used to transform competent XL1-blue cells. Colonies obtained after transformation were screened by colony PCR using gene-specific primers. Mutations were confirmed by DNA sequencing conducted at the LSU College of Science Genomics Facility.

### Membrane preparation and western blotting

Cell membranes was prepared from exponential phase cultures containing indicated plasmid DNA grown under inducing conditions (0.1% arabinose) using a previously published protocol^15,75^. Equal amounts of protein were resolved by SDS-PAGE and transferred to PVDF membrane. Western blotting was carried out using penta-His (Qiagen) primary antibody at 1:5000 dilution and goat-anti-mouse horseradish peroxidase (HRP) conjugated secondary antibody (Pierce) at 1:5000. Detection was performed using the ImmunStar HRP kit (Bio-Rad).

### Susceptibility to colistin and other antibiotics

For testing the susceptibility on solid medium, overnight cultures were freshly diluted 1:100 in MH2 media with appropriate antibiotics and additives, and grown to OD_600_ ~ 0.6 at 37 °C in a shaking incubator. Five microliters of serially log_10_-diluted cells were spotted onto MH2 or LB agar plates containing various concentrations of antibiotic. Growth was analyzed after incubation for 24 h at 37 °C unless otherwise indicated. Antibiotic MIC’s were measured using the broth microdilution method in 96 well plates or using colistin test gradient strips (Biomerieux). All experiments were repeated at least three times.

### Mass spectrometry

For isolation of lipid A, *K. pneumoniae* cultures were grown at 37 °C to an OD_600_ of ~ 1.0. Lipid A chemical extraction was carried out after mild acidic hydrolysis of LPS as previously described^76,77^. For visualization of lipid A by mass spectrometry, lipids were analyzed using MALDI-TOF (ABI 4700 Proteomic Analyzer) in the negative-ion linear mode as previously described^78,79^. Briefly, lipid A samples were dissolved in a mixture of chloroform–methanol (4:1, vol/vol), and 1 µl of sample was mixed with 1 µl of matrix solution. The matrix consisted of 5-chloro-2-mercaptobenzothiazole (CMBT) (20 mg/mL) resuspended in chloroform–methanol-water (4:4:1, vol/vol/vol) mixed with saturated ammonium citrate (20:1, vol/vol). One µl of sample-matrix mixture was loaded on to MALDI target plate for final analysis.

### Measurement of membrane potential

Measurement of membrane potential was performed using JC-1 dye^80^. Briefly, 2.5 × 10^7^ cells from the overnight cultures were inoculated in 20 ml of fresh LB broth in a 250 ml flask and grown for about 1 h 40 min at 37 °C with shaking. ~ 6 × 10^8^ cells were taken from each culture, washed with permeabilization buffer PB, pH 7.5 (10 mM Tris, 1 mM EDTA, 10 mM glucose) and resuspended in fresh PB. 3 µM of JC-1 dye was added and incubated in the dark at 30 °C for 30 min. Cells were washed and resuspended in PB buffer and fluorescence measurements were carried out using a JASCO FP-6300 spectrofluorometer. Relative membrane potential is expressed the ratio of red (595 nm) to green (530 nm) fluorescence with excitation of 488 nm.

### *Galleria mellonella* model of virulence

Larvae purchased from Carolina Biological Supply Company (item# 143,928) were injected with 5 × 10^5^ CFU of bacteria resuspended in 5 µl PBS pH 7.4 or PBS alone. After the injection, larvae were incubated at 37 °C in the dark over a period of 96 h. Larvae were monitored for their survival every 24 h. Dead larvae were identified by the lack of motility or bodily movement against physical stimuli. To measure bacterial survival in *G. mellonella*, larvae were injected with 5 × 10^5^ CFU in PBS pH 7.4 and incubated at 37 °C in the dark. Larvae were homogenized at indicated times and serial dilutions were plated in LB agar containing 100 µg/mL apramycin. No apramycin resistant bacteria were isolated from control larvae injected with PBS under these conditions.

## Supplementary Information

Supplementary Information.

## References

[1] Paterson DL, Harris PN (2016). Colistin resistance: a major breach in our last line of defence. Lancet Infect. Dis..

[2] De Oliveira DMP (2020). Antimicrobial resistance in ESKAPE pathogens. Clin. Microbiol. Rev..

[3] World Health Organization. *Antimicrobial resistance : global report on surveillance 2014*. (World Health Organization, 2014).

[4] Kahler CM, Sarkar-Tyson M, Kibble EA, Stubbs KA, Vrielink A (2018). Enzyme targets for drug design of new anti-virulence therapeutics. Curr. Opin. Struct. Biol..

[5] Harris TL (2014). Small molecule downregulation of PmrAB reverses lipid A modification and breaks colistin resistance. ACS Chem. Biol..

[6] Jana B (2017). The secondary resistome of multidrug-resistant *Klebsiella pneumoniae*. Sci. Rep..

[7] Kline T (2008). Synthesis of and evaluation of lipid A modification by 4-substituted 4-deoxy arabinose analogs as potential inhibitors of bacterial polymyxin resistance. Bioorg. Med. Chem. Lett..

[8] Boughner LA, Doerrler WT (2012). Multiple deletions reveal the essentiality of the DedA membrane protein family in *Escherichia coli*. Microbiology.

[9] Doerrler WT, Sikdar R, Kumar S, Boughner LA (2013). New functions for the ancient DedA membrane protein family. J. Bacteriol..

[10] Liang FT (2010). BB0250 of *Borrelia burgdorferi* is a conserved and essential inner membrane protein required for cell division. J. Bacteriol..

[11] Sikdar R, Doerrler WT (2010). Inefficient Tat-dependent export of periplasmic amidases in an *Escherichia coli* strain with mutations in two DedA family genes. J. Bacteriol..

[12] Sikdar R, Simmons AR, Doerrler WT (2013). Multiple envelope stress response pathways are activated in an *Escherichia coli* strain with mutations in two members of the DedA membrane protein family. J. Bacteriol..

[13] Thompkins K, Chattopadhyay B, Xiao Y, Henk MC, Doerrler WT (2008). Temperature sensitivity and cell division defects in an *Escherichia coli* strain with mutations in *yghB* and *yqjA*, encoding related and conserved inner membrane proteins. J. Bacteriol..

[14] Kumar S, Bradley CL, Mukashyaka P, Doerrler WT (2016). Identification of essential arginine residues of *Escherichia coli* DedA/Tvp38 family membrane proteins YqjA and YghB. FEMS Microbiol. Lett..

[15] Kumar S, Doerrler WT (2014). Members of the conserved DedA family are likely membrane transporters and are required for drug resistance in *Escherichia coli*. Antimicrob. Agents Chemother..

[16] Kumar S, Doerrler WT (2015). *Escherichia coli* YqjA, a member of the conserved DedA/Tvp38 membrane protein family, is a putative osmosensing transporter required for growth at alkaline pH. J. Bacteriol..

[17] Panta PR, Doerrler WT (2021). A *Burkholderia thailandensis* DedA family membrane protein is required for proton motive force dependent lipid a modification. Front. Microbiol..

[18] Panta PR (2019). A DedA family membrane protein is required for *Burkholderia thailandensis* Colistin Resistance. Front. Microbiol..

[19] Iqbal A (2021). Chemical or genetic alteration of proton motive force results in loss of virulence of *Burkholderia glumae*, the cause of rice bacterial panicle blight. Appl. Environ. Microbiol..

[20] Panta PR, Doerrler WT (2021). A link between pH homeostasis and colistin resistance in bacteria. Sci. Rep..

[21] Gerchman Y (1993). Histidine-226 is part of the pH sensor of NhaA, a Na^+^/H^+^ antiporter in *Escherichia coli*. Proc. Natl. Acad. Sci. U S A.

[22] Noumi T, Inoue H, Sakurai T, Tsuchiya T, Kanazawa H (1997). Identification and characterization of functional residues in a Na^+^/H^+^ antiporter (NhaA) from *Escherichia coli* by random mutagenesis. J. Biochem..

[23] Adler J, Bibi E (2004). Determinants of substrate recognition by the *Escherichia coli* multidrug transporter MdfA identified on both sides of the membrane. J. Biol. Chem..

[24] Sigal N (2005). 3D model of the *Escherichia coli* multidrug transporter MdfA reveals an essential membrane-embedded positive charge. Biochemistry.

[25] Holdsworth SR, Law CJ (2012). Functional and biochemical characterisation of the *Escherichia coli* major facilitator superfamily multidrug transporter MdtM. Biochimie.

[26] Fluman N, Ryan CM, Whitelegge JP, Bibi E (2012). Dissection of mechanistic principles of a secondary multidrug efflux protein. Mol. Cell.

[27] Abramson J, Iwata S, Kaback HR (2004). Lactose permease as a paradigm for membrane transport proteins (Review). Mol. Membr. Biol..

[28] Cain BD, Simoni RD (1989). Proton translocation by the F1F0ATPase of *Escherichia coli*. Mutagenic analysis of the a subunit. J. Biol. Chem..

[29] Hellmer J, Teubner A, Zeilinger C (2003). Conserved arginine and aspartate residues are critical for function of MjNhaP1, a Na^+^/H^+^ antiporter of *M. jannaschii*. FEBS Lett.

[30] Shi Y, Cromie MJ, Hsu FF, Turk J, Groisman EA (2004). PhoP-regulated *Salmonella* resistance to the antimicrobial peptides magainin 2 and polymyxin B. Mol. Microbiol..

[31] Tzeng YL (2005). Cationic antimicrobial peptide resistance in *Neisseria meningitidis*. J. Bacteriol..

[32] Poirel L, Jayol A, Nordmann P (2017). Polymyxins: antibacterial activity, susceptibility testing, and resistance mechanisms encoded by plasmids or chromosomes. Clin. Microbiol. Rev..

[33] Yeaman MR, Yount NY (2007). Unifying themes in host defence effector polypeptides. Nat. Rev. Microbiol..

[34] Lazzaro BP, Zasloff M, Rolff J (2020). Antimicrobial peptides: application informed by evolution. Science.

[35] Raetz CR, Reynolds CM, Trent MS, Bishop RE (2007). Lipid A modification systems in gram-negative bacteria. Annu. Rev. Biochem..

[36] Liu YY (2016). Emergence of plasmid-mediated colistin resistance mechanism MCR-1 in animals and human beings in China: a microbiological and molecular biological study. Lancet Infect. Dis..

[37] McGann P (2016). *Escherichia coli* Harboring *mcr-1* and *blaCTX-M* on a Novel IncF Plasmid: First Report of *mcr-1* in the United States. Antimicrob. Agents Chemother..

[38] Rolain JM (2016). Plasmid-Mediated *mcr-1* Gene in Colistin-Resistant Clinical Isolates of *Klebsiella pneumoniae* in France and Laos. Antimicrob. Agents Chemother..

[39] Alteri CJ, Lindner JR, Reiss DJ, Smith SN, Mobley HL (2011). The broadly conserved regulator PhoP links pathogen virulence and membrane potential in Escherichia coli. Mol. Microbiol..

[40] Insua JL (2013). Modeling Klebsiella pneumoniae pathogenesis by infection of the wax moth Galleria mellonella. Infect. Immun..

[41] Wright MS (2015). Genomic and transcriptomic analyses of colistin-resistant clinical isolates of Klebsiella pneumoniae reveal multiple pathways of resistance. Antimicrob. Agents Chemother..

[42] Leung LM (2017). Structural modification of LPS in colistin-resistant, KPC-producing Klebsiella pneumoniae. J. Antimicrob. Chemother..

[43] Lee DD (2019). Magnesium flux modulates ribosomes to increase bacterial survival. Cell.

[44] Vaara M (1992). Agents that increase the permeability of the outer membrane. Microbiol. Rev..

[45] Vescovi EG, Soncini FC, Groisman EA (1996). Mg^2+^ as an extracellular signal: environmental regulation of Salmonella virulence. Cell.

[46] Petrou VI (2016). Structures of aminoarabinose transferase ArnT suggest a molecular basis for lipid A glycosylation. Science.

[47] Tavares-Carreon F, Fathy Mohamed Y, Andrade A, Valvano MA (2016). ArnT proteins that catalyze the glycosylation of lipopolysaccharide share common features with bacterial N-oligosaccharyltransferases. Glycobiology.

[48] Trent MS, Ribeiro AA, Lin S, Cotter RJ, Raetz CR (2001). An inner membrane enzyme in Salmonella and Escherichia coli that transfers 4-amino-4-deoxy-L-arabinose to lipid A: induction on polymyxin-resistant mutants and role of a novel lipid-linked donor. J. Biol. Chem..

[49] Simon R, Priefer U, Puhler A (1983). A broad host range mobilization system for invivo genetic-engineering - transposon mutagenesis in gram-negative bacteria. Bio-Technol..

[50] Datsenko KA, Wanner BL (2000). One-step inactivation of chromosomal genes in *Escherichia coli* K-12 using PCR products. Proc. Natl. Acad. Sci. U S A.

[51] Cardona ST, Valvano MA (2005). An expression vector containing a rhamnose-inducible promoter provides tightly regulated gene expression in *Burkholderia cenocepacia*. Plasmid.

[52] Tsai CJ, Loh JM, Proft T (2016). Galleria mellonella infection models for the study of bacterial diseases and for antimicrobial drug testing. Virulence.

[53] Koch-Weser J (1970). Adverse effects of sodium colistimethate. Manifestations and specific reaction rates during 317 courses of therapy. Ann. Int. Med..

[54] Hussein NH, Al-Kadmy IMS, Taha BM, Hussein JD (2021). Mobilized colistin resistance (mcr) genes from 1 to 10: a comprehensive review. Mol. Biol. Rep..

[55] Olaitan AO, Morand S, Rolain JM (2014). Mechanisms of polymyxin resistance: acquired and intrinsic resistance in bacteria. Front. Microbiol..

[56] Needham BD, Trent MS (2013). Fortifying the barrier: the impact of lipid A remodelling on bacterial pathogenesis. Nat. Rev. Microbiol..

[57] Sabnis A (2021). Colistin kills bacteria by targeting lipopolysaccharide in the cytoplasmic membrane. Elife.

[58] Khafizov K, Staritzbichler R, Stamm M, Forrest LR (2010). A study of the evolution of inverted-topology repeats from LeuT-fold transporters using AlignMe. Biochemistry.

[59] Keller R, Ziegler C, Schneider D (2014). When two turn into one: evolution of membrane transporters from half modules. Biol. Chem..

[60] Mesdaghi S, Murphy DL, Sanchez Rodriguez F, Burgos-Marmol JJ, Rigden DJ (2020). In silico prediction of structure and function for a large family of transmembrane proteins that includes human Tmem41b. F1000Res.

[61] Shoemaker CJ (2019). CRISPR screening using an expanded toolkit of autophagy reporters identifies TMEM41B as a novel autophagy factor. PLoS Biol..

[62] Morita K, Hama Y, Mizushima N (2019). TMEM41B functions with VMP1 in autophagosome formation. Autophagy.

[63] Morita K (2018). Genome-wide CRISPR screen identifies TMEM41B as a gene required for autophagosome formation. J. Cell Biol..

[64] Hoffmann HH (2021). TMEM41B is a pan-flavivirus host factor. Cell.

[65] Schneider WM (2021). Genome-Scale Identification of SARS-CoV-2 and Pan-coronavirus Host Factor Networks. Cell.

[66] Tabara LC, Vincent O, Escalante R (2019). Evidence for an evolutionary relationship between Vmp1 and bacterial DedA proteins. Int. J. Dev. Biol..

[67] Pereira MF, Rossi CC, da Silva GC, Rosa JN, Bazzolli DMS (2020). Galleria mellonella as an infection model: an in-depth look at why it works and practical considerations for successful application. Pathog. Dis..

[68] Browne N, Heelan M, Kavanagh K (2013). An analysis of the structural and functional similarities of insect hemocytes and mammalian phagocytes. Virulence.

[69] Mills G, Dumigan A, Kidd T, Hobley L, Bengoechea JA (2017). Identification and Characterization of Two Klebsiella pneumoniae lpxL Lipid A Late Acyltransferases and Their Role in Virulence. Infect. Immun..

[70] Seed KD, Dennis JJ (2008). Development of Galleria mellonella as an alternative infection model for the Burkholderia cepacia complex. Infect. Immun..

[71] Asai M (2019). Galleria mellonella: an infection model for screening compounds against the Mycobacterium tuberculosis complex. Front. Microbiol..

[72] Huang TW (2014). Capsule deletion via a lambda-Red knockout system perturbs biofilm formation and fimbriae expression in Klebsiella pneumoniae MGH 78578. BMC Res. Notes.

[73] Simon R, Priefer U, Pühler A (1983). A broad host range mobilization system for in vivo genetic engineering: transposon mutagenesis in gram negative bacteria. Bio/Technology.

[74] Zheng L, Baumann U, Reymond JL (2004). An efficient one-step site-directed and site-saturation mutagenesis protocol. Nucleic Acids Res..

[75] Doerrler WT, Raetz CR (2002). ATPase activity of the MsbA lipid flippase of *Escherichia coli*. J. Biol. Chem..

[76] Zhou Z, Lin S, Cotter RJ, Raetz CR (1999). Lipid A modifications characteristic of Salmonella typhimurium are induced by NH_4_VO_3_ in Escherichia coli K12. Detection of 4-amino-4-deoxy-L-arabinose, phosphoethanolamine and palmitate. J. Biol. Chem..

[77] Herrera CM (2014). The *Vibrio cholerae* VprA-VprB two-component system controls virulence through endotoxin modification. MBio.

[78] Henderson JC, O'Brien JP, Brodbelt JS, Trent MS (2013). Isolation and chemical characterization of lipid A from gram-negative bacteria. J. Vis. Exp..

[79] Zhou P, Altman E, Perry MB, Li J (2010). Study of matrix additives for sensitive analysis of lipid A by matrix-assisted laser desorption ionization mass spectrometry. Appl. Environ. Microbiol..

[80] Panta PR (2019). A DedA family membrane protein is required for Burkholderia thailandensis colistin resistance. Front. Microbiol..

