# Supplementary material for: A Klebsiella pneumoniae DedA family membrane protein is required for colistin resistance and for virulence in wax moth larvae
Source: Sci Rep. 2021 Dec 21;11:24365. doi: 10.1038/s41598-021-03834-3 (PMC8692421; doi:10.1038/s41598-021-03834-3)
Supplement: Supplementary file 1 — Supplementary Information. [file 41598_2021_3834_MOESM1_ESM.pdf]

Supplemental material for:

A *Klebsiella pneumoniae* DedA family membrane protein is required for colistin resistance and for virulence in wax moth larvae

Vijay Tiwari[1], Pradip R. Panta[1], Caitlin E. Billiot[1], Martin V. Douglass[2], Carmen M. Herrera[2], M. Stephen Trent[2], and William T. Doerrler[1*]

Affiliations:
[1]Department of Biological Sciences, Louisiana State University, Baton Rouge, LA, USA

[2]Department of Infectious Diseases, University of Georgia, College of Veterinary Medicine, Athens, GA, USA

*** Correspondence:**
William T. Doerrler
wdoerr@lsu.edu

```
CLUSTAL O (1.2.4) multiple sequence alignment


EcYghB       ---------------------------MAVIQDIIAALWQHDF-AALADPHIVSVVYFVM   32
EcYqjA       ---------------------------MELLTQLLQALWAQDF-ETLANPSMIGMLYFVL   32
KpnDkcA      ---------------------------MDLIHFLIDFILHIDVHLAELVAQYGVWVYAIL   33
EcDedA       ---------------------------MDLIYFLIDFILHIDVHLAELVAEYGVWVYAIL   33
BthDbcA      MTALLFLLPAPLRPARAHFVTDTKETHLETLLHFVSLVVHIDAFLGDFIRQYGAWVYLVL   60
                                        :  :  ::  :   *             :* ::

EcYghB       FATLFLENGLLPASFLPGDSLLILAGALIAQG--VMDFLPTIAILTAAASLGCWLSYIQG   90
EcYqjA       FVILFLENGLLPAAFLPGDSLLVLVGVLIAKG--AMGYPQTILLLTVAASLGCWVSYIQG   90
KpnDkcA      FLILFCETGLVVTPFLPGDSLLFVAGALSALPTNDLNVHLMVLLMVVAAIVGDAVNYTIG   93
EcDedA       FLILFCETGLVVTPFLPGDSLLFVAGALASLETNDLNVHMMVVLMLIAAIVGDAVNYTIG   93
BthDbcA      FLIVFCETGLVIFPFLPGDSLLFIAGAFAATG--EMTLAGLIVLLLVAAVGGNTVNYLIG   118
             *  :* *.**:   ********.:.*.: :     :     : ::  **  *  :.*  *

EcYghB       RWLGNTKTV---KGWLAQLPAKYHQRATCMFDRHGLLALLAGRFLAFVRTLLPTMAGISG   147
EcYqjA       RWLGNTRTV---QNWLSHLPAHYHQRAHHLFHKHGLSALLIGRFIAFVRTLLPTIAGLSG   147
KpnDkcA      RLFGEKLFSNPNS---KIFRRSYLDKTHSFYERHGGKTIILARFVPIVRTFAPFVAGMGH   150
EcDedA       RLFGEKLFSNPNS---KIFRRSYLDKTHQFYEKHGGKTIILARFVPIVRTFAPFVAGMGH   150
BthDbcA      RAIGPKVFNTHIPGLERFLDRAALLKTHNFYERHGGKTLVLARFIPVVRTFAPFVAGASA   178
             * :* .            :      ::  ::.:**  ::: .**: .***: * :** .

EcYghB       LPNRRFQFFNWLSGLLWVSVVTSFGYALSMIPFVKRHEDQVMTFLMILPIALLTAGLLGT   207
EcYqjA       LNNARFQFFNWMSGLLWVLILTTLGYMLGKTPVFLKYEDQLMSCLMLLPVVLLVFGLAGS   207
KpnDkcA      MSYRHFAAYNVVGALLWVLLFTYAGYLFGDLPVVQENLKLLIVAIIVLS---VLPGVIE-   206
EcDedA       MSYRHFAAYNVIGALLWVLLFTYAGYFFGTIPMVQDNLKLLIVGIIVVS---ILPGVIE-   206
BthDbcA      MRFARFQLFNVIGALIWVLLLVLLGYFFGNIPFIRHYLNVIVLVGIGAA---VIPVAIGA   235
             :   :*  :* :..*:** :..  ** :.  *..    . ::   :      :

EcYghB       LFVVIKKKYCNA------   219
EcYqjA       LVVLWKKKYGNRG-----   220
KpnDkcA      ---IIRHKRAAAKQAK--   219
EcDedA       ---IIRHKRAAARAAK--   219
BthDbcA      LWKLLRRKPGAQKTQANR   253
                : ::*
```

Fig S1. Amino acid alignment of select bacterial homologs of *Klebsiella pneumoniae* DkcA. Functional acidic (E39, D51; YqjA numbering) and basic (R130, R136; YqjA numbering) amino acids are in bold font. Abbreviations: Ec, *Escherichia coli*; Kpn, *Klebsiella pneumoniae*; Bth, *Burkholderia thailandensis*. Clustal Omega [1] was used to generate alignment.

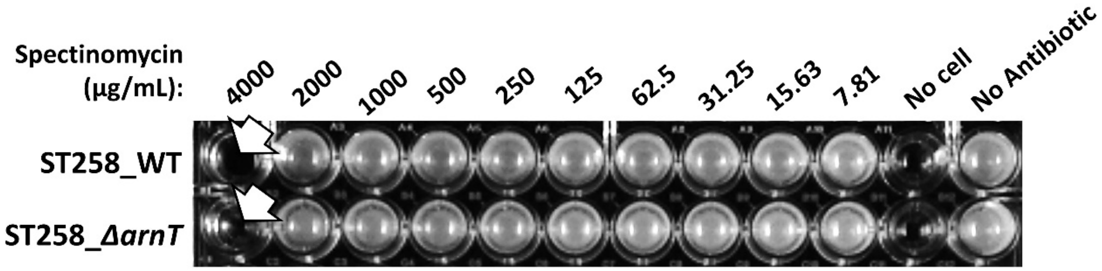

Fig S2. Measurement of spectinomycin MIC for Δ*arnT* strain. Spectinomycin MIC was measured using wild type (*K. pneumoniae* ST258) and Δ*arnT*. Approximate MIC is indicated with arrows.

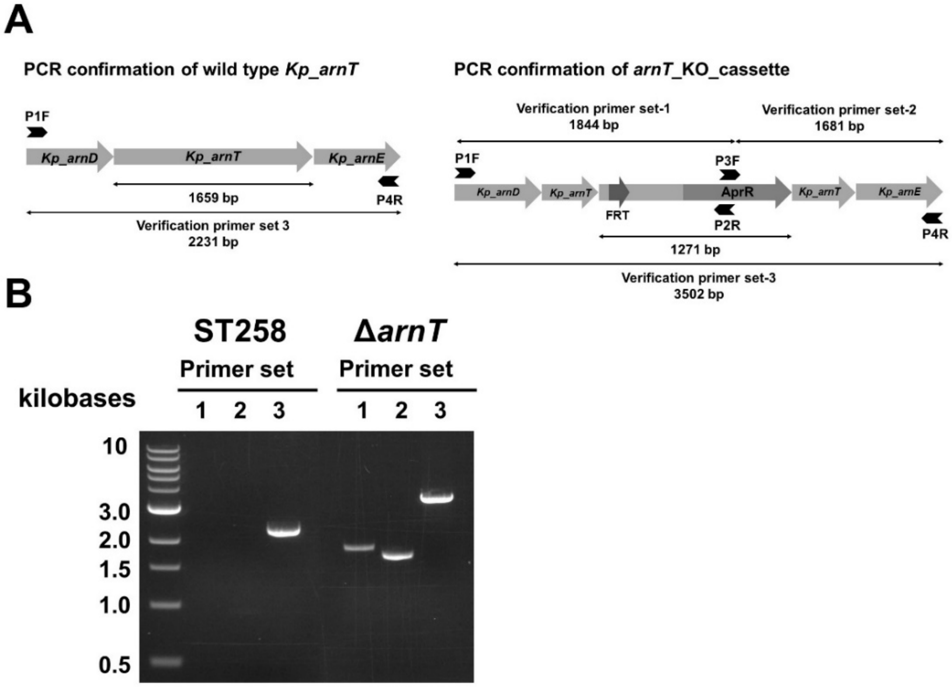

Fig S3. Construction of *K. pneumoniae* ST258 Δ*arnT*. (A) Diagram showing *arnT* with its adjacent genes, *arnD* and *arnE*. P1F, P2R, P3F and P4R are used for the verification of insertion of the knockout cassette. Genes are not drawn to scale. **(B)** Ethidium bromide stained agarose gel with products of PCR reactions using three verification primer sets; primer set 1 (P1F & P2R), primer set 2 (P3F & P4R) and primer set 3 (P1F & P4R).

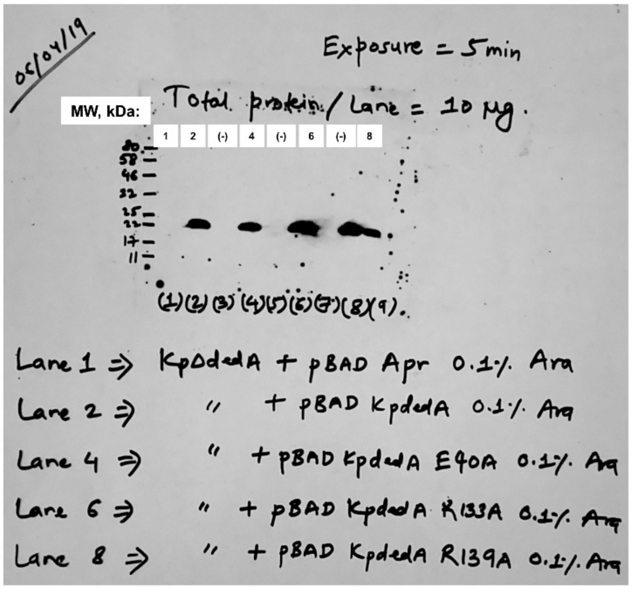

Fig. S4. Expression of *dkcA* and point mutants in membrane fractions as determined by Western blotting with anti-hexahistidine antibody (non-cropped version of Figure 1D). The image was not manipulated in any way. Abbreviations: (-) is lane with no protein loaded. Ten µg of membrane protein was loaded per lane and strains were grown in the presence of 50 µg/ml apramycin and 0.1% arabinose.

Reference:

1 Sievers, F. & Higgins, D. G. Clustal Omega, accurate alignment of very large numbers of sequences. *Methods Mol Biol* **1079**, 105-116, doi:10.1007/978-1-62703-646-7_6 (2014).
